# Multiple types of distress are prospectively associated with increased risk of ovarian cancer

**DOI:** 10.1002/cam4.6125

**Published:** 2023-06-16

**Authors:** Andrea L. Roberts, Andrew Ratanatharathorn, Lori Chibnik, Laura D. Kubzansky, Shelley S. Tworoger

**Affiliations:** ^1^ Department of Environmental Health Harvard T. H. Chan School of Public Health Boston Massachusetts USA; ^2^ Department of Cancer Epidemiology Moffitt Cancer Center Tampa Florida USA

**Keywords:** anxiety, depression, distress, epidemiology, ovarian cancer, posttraumatic stress disorder, PTSD, social isolation, widowhood

## Abstract

**Background:**

Few modifiable risk factors for epithelial ovarian cancer have been identified. We and other investigators have found that individual psychosocial factors related to distress are associated with higher risk of ovarian cancer. The present study examined whether co‐occurring distress‐related factors are associated with ovarian cancer risk.

**Methods:**

Five distress‐related factors were measured repeatedly over 21 years of follow‐up: depression, anxiety, social isolation, widowhood, and, in a subset or women, posttraumatic stress disorder (PTSD). Cox proportional hazards models estimate relative risks (RR) and 95% confidence intervals (CI) of ovarian cancer for a time‐updated count of distress‐related factors, in age‐adjusted models, then further adjusted for ovarian cancer risk factors and behavior‐related health risk factors.

**Results:**

Across 1,193,927 person‐years of follow‐up, 526 incident ovarian cancers occurred. Women with ≥3 versus no distress‐related psychosocial factors demonstrated increased ovarian cancer risk (HR_age‐adjusted_ = 1.71; 95% CI = 1.16, 2.52). No significant difference in ovarian cancer risk was observed in women with one or two versus no distress‐related psychosocial factors. In the subsample with PTSD assessed, ≥3 versus no distress‐related psychosocial factors was associated with twofold greater ovarian cancer risk (HR_age‐adjusted_ = 2.08, 95% CI = 1.01, 4.29). Further analysis suggested that women at highest ovarian cancer risk had PTSD co‐occurring with any other distress‐related factor (HR = 2.19, 95% CI = 1.20, 4.01). Adjusting for cancer risk factors and health behaviors minimally impacted risk estimates.

**Conclusions:**

Presence of multiple indicators of distress was associated with risk of ovarian cancer. When including PTSD as an indicator of distress, the association was strengthened.

## Graphical abstract

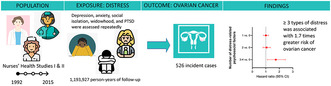

We assessed 5 types of distress in two large cohorts of women across up to 23 years. Experience of three or more types of distress was associated with 1.7 times greater risk of incident ovarian cancer. Adjusting for health behaviors and ovarian cancer risk factors had a minimal impact on risk estimates.

## 1 INTRODUCTION

Epithelial ovarian cancer is the fifth leading cause of female cancer death in the United States.^1^ However, few modifiable risk factors have been identified. Psychological stress has been linked to faster ovarian cancer tumor growth, increased invasiveness, and evasion of apoptosis in animal and in vitro models.^2^, ^3^, ^4^ Stress has been linked to multiple biological processes involved in ovarian tumor formation, growth, and invasiveness, including DNA damage and damage repair,^5^ inflammation, angiogenesis, cell motility, and cellular immune response.^6^, ^7^, ^8^

Individually, various psychosocial factors that are characterized by or commonly result in distress including depression,^9^, ^10^ anxiety,^11^ posttraumatic stress disorder (PTSD),^12^ and social isolation^13^ have been associated with a higher risk of developing ovarian cancer in prospective studies, including in our own research, though not uniformly.^14^ Studies of antidepressant use have had mixed findings,^15^ perhaps because these medications reduce the biologic impact of distress on carcinogenesis.

Importantly, prior work examining individual psychosocial factors may have misestimated the relationship of distress with cancer risk because different forms of distress (e.g., anxiety, depression) frequently co‐occur, which could lead to confounding by unmeasured forms of distress or misclassification of individuals in the reference group—who may have been characterized as having no distress when in fact they had other forms of distress. For example, if anxiety has a causal effect on cancer incidence, studies of depression that do not account for anxiety would misestimate the association of depression with cancer incidence given the co‐morbidity of these conditions. Meta‐analyses of the relation between depression and cancer incidence have noted that studies typically account for few potential confounders and rarely account for other mental health symptoms or sources of distress.^16^, ^17^ Moreover, recent investigations of other diseases suggest that the combined effects of multiple forms of psychosocial distress and distress‐related factors may be of greater magnitude than associations observed with any single form.^18^ Experiencing multiple forms of distress, versus only a single form, could signify higher levels or more persistent distress that more strongly affect biological and behavioral dysregulation relevant for disease risk.

Thus, in the present study, we prospectively examined the combination of multiple forms of distress, including depression, anxiety, and PTSD, and experiences commonly associated with high levels of distress, including social isolation and widowhood, in relation to risk of developing ovarian cancer. We use two large prospective cohorts of women, the Nurses' Health Study (NHS) and the Nurses' Health Study II (NHSII) and examine risk overall, by menopausal status, and separately for high‐grade serous/poorly differentiated cancers, a common and aggressive form of the disease.

Relevant psychosocial factors were selected based on prior work, including in NHS and NHSII, demonstrating increased risk of incident ovarian cancer with each factor examined separately.^9^, ^11^, ^12^, ^13^ In these prior analyses, PTSD was more strongly associated with ovarian cancer than were other types of distress. Therefore, in the current study we also investigated the extent to which co‐occurring PTSD may account for the association of other types of distress with incident ovarian cancer risk.

## 2 METHODS

### 2.1 Sample

The NHS is an ongoing longitudinal study of 121,700 female nurses enrolled in 1976 at ages 30–55 years. The NHSII is a companion study of 116,429 female nurses aged 24–42 years begun in 1989. Both studies queried participants at baseline about lifestyle factors, health behaviors, and medical history via questionnaire. Biennial and additional one‐time questionnaires were used to update health status and distress measures over follow‐up. The study protocol was approved by the institutional review boards of the Brigham and Women's Hospital and Harvard T.H. Chan School of Public Health, and those of participating registries as required. Return of questionnaires by mail constituted implied consent. Signed releases were obtained to collect medical records.

### 2.2 Distress‐related psychosocial factors

Five distress‐related psychosocial factors were measured repeatedly over follow‐up, including depression, anxiety, social isolation, widowhood, and, in a subset of participants, PTSD.

The five‐item Mental Health Index (MHI‐5) assessed depressive symptoms (NHS: 1992, 1996, 2000; NHS2: 1993, 1997, 2001).^19^ Women were asked how much of the time during the past 4 weeks they felt: nervous; so down that nothing could cheer them up; calm and peaceful; down and blue; or happy, with possible responses ranging from “none” to “all.” Responses were scored from 0 to 100, with a score ≤60 indicating moderate or severe depressive symptoms.^20^ Past‐two‐year antidepressant use was queried starting in 1996 (NHS) and 1993 (NHSII). Participants reported whether they had clinician‐diagnosed depression in the previous 2 years, starting in 2000 (NHS) and 2003 (NHS2). Participants were categorized as having probable depression if they scored ≤60 on the MHI‐5,^9^, ^20^ reported using antidepressants, or had a depression diagnosis.

Symptoms of anxiety were assessed using the eight‐item Crown‐Crisp phobic anxiety index (CCI) (NHS: 1988, 2004; NHSII: 1993, 2005).^21^ Scores ranged from 0 to 16, with participants scoring ≥4 classified as having high anxiety.^22^ Psychosocial factors associated with high levels of distress, for example, social isolation and self‐reported widowhood, were assessed every 4 years beginning in 1992 (NHS) and 2001 (NHSII). Social isolation was assessed using the Berkman–Syme Social Network Index (SNI), which assessed four types of social connections: (1) marital status (married or unmarried), (2) close friends and relatives, (3) religious participation, and (4) nonreligious group participation.^23^ Following prior work in the cohort, women were considered isolated if they reported having 0 or 1 type of social connection.^13^

Every 2 years, we created a time‐updated count of number of distress‐related psychosocial factors based on the most recent data reported for each factor. As few women reported all four types of distress, our highest level included participants who reported three or four types.

To examine the association of duration of distress‐related factors with ovarian cancer, we coded the number of distress‐related factors across each 4 years of follow‐up as: none (reference); high (3 or 4 in both 2‐year periods); or mixed (at least one in one of the two sequential 2‐year periods, but fewer than 3 or 4 in both 2‐year periods).

In a subsample of NHSII women, information on trauma exposure and PTSD was available. In 2008, lifetime exposure to trauma was assessed using the Brief Trauma Questionnaire.^24^ Participants were asked the age at which their worst traumatic event occurred. Lifetime PTSD symptoms were queried in relation to this event using a seven‐item scale.^25^ Women with ≥4 PTSD symptoms were considered to have probable PTSD in the year of their worst event and subsequently. For this subsample, we calculated a second measure of number of types of distress including five factors: depression, anxiety, widowhood, isolation, and PTSD. As few participants reported more than three types of distress, we combined those reporting three to five types into a single level.

### 2.3 Ovarian cancer

Ovarian cancer diagnosis was self‐reported on each biennial questionnaire with follow‐up through 2015. We identified additional cases through report by family members and via the National Death Index. We obtained an adjudicated indicator by requesting pathology reports or linking to the relevant cancer registry, to obtain information by which a gynecologic pathologist, blinded to women's exposure status, abstracted information on morphology, histology, stage, and grade. In a sample of 459 ovarian cancer cases, concordance between a centralized review of slides and pathology report abstraction was 78% for histologic type, 79% for grade, and 94% for invasiveness.^26^

### 2.4 Covariates

Ovarian cancer risk factors and relevant health risk factors were queried regularly on the biennial questionnaires and, for the current study, were time‐updated approximately every 2–4 years. These included duration of oral contraceptive use (never, <1, 1–5, >5 years), history of tubal ligation or hysterectomy (ever/never), family history of ovarian cancer (any/none), menopausal status (pre‐ or postmenopausal), parity (coded continuously), smoking status (never, past, or current), body mass index (BMI; <18, 18–25, 25–30, or >30 kg/m^2^), and past‐year leisure‐time physical activity (<3, 3–8.9, 9–17.9, 18–26.9, or >27 metabolic equivalent hours/week). Self‐report of health‐related factors has good validity in the NHS cohorts.^27^, ^28^

### 2.5 Analyses

Follow‐up began in the first year by which all distress types had been assessed (NHS: 1994; NHSII: 2003, referred to henceforth as baseline). Participants were included in analyses if they completed each first assessment of the distress‐related psychosocial factors (NHS: 1988 and 1994; NHSII: 2001 and 2003), had not been diagnosed with cancer at the time of these assessments, and had not had a bilateral oophorectomy or menopause due to pelvic irradiation prior to each respective baseline. After exclusions, 65,066 NHS and 50,628 NHSII participants were included. Participants accrued person‐time from baseline until the date of ovarian cancer diagnosis (cases), removal of ovaries, pelvic radiation, breast cancer diagnosis, death, non‐responsiveness to biennial questionnaires, or end of follow‐up in 2019 (non‐cases), whichever came first.

We examined the distribution of covariates across the number of distress‐related factors in each sample. We then used Cox proportional hazards models to estimate relative risks (RR) and 95% confidence intervals (CI) of ovarian cancer for each time‐updated count of distress‐related factors, separately by cohort. To reduce the likelihood that ovarian cancer symptoms might have induced distress, we included a latency period of 4–6 years. For example, we examined distress‐related factors from 2002 to 2004 and risk of ovarian cancer from 2006 to 2008. We assessed statistical heterogeneity across the two cohorts using meta‐analysis, and, finding no substantive difference (*I* ^2^ < 20%),^29^ we pooled the data. We fit a series of models: (1) stratified by age, calendar time in months, and cohort, to allow for differences in baseline hazard; (2) further adjusted for time‐updated ovarian cancer risk factors, including oral contraceptive use, tubal ligation, history of hysterectomy, family history of breast cancer, use and duration of postmenopausal hormones, menopause status, and parity; (3) including Model 2 covariates, and further adjusted for time‐updated behavior‐related health risk factors, including BMI, physical activity, and smoking, which may act as mediators. To determine whether there was a linear association with increasing number of distress‐related factors, we tested the trend across number of factors as a continuous variable, ranging from 0 to 3, with three representing ≥3. We additionally examined risk of ovarian cancer in relation to longer lasting distress‐related factors by fitting a model with distress across 4 years as the independent variable.

We conducted a secondary analysis in the subsample of NHSII participants who provided information on PTSD. First, to directly compare associations in this subsample with those in the main analyses, we examined number of distress‐related psychosocial factors, excluding PTSD, with ovarian cancer risk. Next, we added PTSD to the count of psychosocial factors and examined risk of ovarian cancer. Finally, as prior work in this cohort has demonstrated PTSD is strongly associated with ovarian cancer incidence,^12^ we further ascertained risk of ovarian cancer and distress‐related factors (i.e., depression, widowhood, social isolation, and phobic anxiety) in the presence or absence of PTSD, using the following categories: (1) no PTSD and no other distress‐related factor [reference], (2) no PTSD and ≥1 other factor; (3) PTSD and no other factors; (4) PTSD and ≥1 other factor. Finally, we examined risk of ovarian cancer in models further adjusted for time‐updated lifetime trauma exposure (any/none) and in models restricted to trauma‐exposed participants.

Sensitivity analyses examined risk of Type 2 ovarian cancers only (i.e., high grade serous/poorly differentiated). For these analyses, women with other types of ovarian cancer were censored at the date of their cancer diagnosis. As cancer risk factors vary by menopausal status, we also conducted analyses stratified by menopausal status using multiplicative interaction terms and a Wald test to assess the *p*‐value for heterogeneity.

For all analyses, inverse probability weighting was used to adjust for the probability of each participant surviving from cohort enrollment until study baseline. Weights were calculated using variables available at enrollment that predicted survival, including age, BMI, height, exercise, pack‐years of cigarette smoking, alcohol consumption, body shape at age 5, cancer history, occurrence of menopause, ovary removal, use of beta blockers, parity, race, and ethnicity.

## 3 RESULTS

At baseline, dose‐dependent relations were observed between number of distress‐related psychosocial factors and current smoking, low physical activity, higher BMI, family history of breast cancer, being post‐menopausal, and never using oral contraceptives (Table 1).

**TABLE 1:** Ovarian cancer risk factors and health‐related risk factors by number of distress‐related psychosocial factors, Nurses' Health Study (1994) and Nurses' Health Study II (2003), *N* = 115,694.

		NHS				NHSII			
		Number of distress‐related psychosocial factors				Number of distress‐related psychosocial factors			
		0	1	2	3–4	0	1	2	3–4
Participants	*N*	31,432	24,219	7584	1831	19,671	19,305	9858	1794
Age, years	Mean (SD)	61.4 (7.4)	62.4 (7.8)	63.3 (8.2)	64.9 (8.4)	48.3 (4.7)	48.7 (4.6)	49 (4.6)	49.4 (4.6)
Race, White	% (*N*)	94.9 (29818)	93.5 (22634)	93.2 (7070)	93.7 (1715)	95.8 (18846)	95 (18335)	94.5 (9314)	93.6 (1679)
Family history of breast or ovarian cancer	% (*N*)	15.1 (4754)	15.5 (3765)	16.2 (1226)	17 (312)	13.3 (2625)	14.1 (2723)	14.6 (1444)	14.4 (258)
Parity, none	% (*N*)	5.3 (1664)	5.2 (1250)	5.7 (435)	5.4 (99)	15.2 (2994)	19.5 (3773)	21.2 (2092)	29.2 (523)
Tubal ligation, ever	% (*N*)	21.6 (6774)	20.5 (4954)	20 (1520)	19.4 (355)	19.6 (3861)	21.1 (4067)	20.2 (1992)	21.4 (384)
Oral contraceptive use, never	% (*N*)	50.1 (15735)	47.5 (11499)	46 (3492)	41.9 (767)	14.7 (2888)	12.8 (2472)	12.8 (1262)	11.2 (201)
Menopause status, postmenopausal	% (*N*)	83.9 (26385)	85.7 (20754)	87 (6599)	90.9 (1665)	24.4 (4798)	27.1 (5237)	29.7 (2923)	32.6 (584)
Hormone use among postmenopausal women, any	% (*N*)	41.7 (10998)	38.8 (8044)	35.6 (2347)	31.4 (522)	37.6 (1802)	41.5 (2171)	41.6 (1217)	38.2 (223)
BMI	Mean (SD)	26.1 (4.9)	26.5 (5.2)	26.8 (5.5)	27.1 (5.7)	25.9 (5.6)	26.9 (6.3)	27.8 (6.8)	28.4 (7.4)
Physical activity, <3 metabolic equivalent hours/week	% (*N*)	14.3 (4489)	17.1 (4151)	21.5 (1633)	28.5 (522)	11.6 (2282)	14.5 (2790)	16.6 (1638)	20.8 (373)
Smoking, current	% (*N*)	10.0 (3154)	13.1 (3161)	18 (1362)	22.6 (413)	4.9 (971)	7.7 (1492)	10.8 (1062)	17.1 (307)

In the two cohorts, 526 incident ovarian cancers occurred (NHS = 403; NHSII = 123) across 1,193,927 person‐years of follow‐up. Women with ≥3 versus no distress‐related psychosocial factors demonstrated significantly increased ovarian cancer risk (HR_age‐adjusted_ = 1.71; 95% CI = 1.16, 2.52; Table 2, Model 1). Further adjustment for ovarian cancer risk factors (HR = 1.66; 95% CI = 1.13, 2.46; Table 2, Model 2), and health behaviors (HR = 1.64; 95% CI = 1.10, 2.44; Table 2, Model 3) did not substantially attenuate this association. No significant difference in ovarian cancer risk was observed in women with one or two versus no distress‐related psychosocial factors. The *p*_trend_ for increasing number of distress‐related psychosocial factors did not reach statistical significance (*p* = 0.11). Having persistently high distress‐related factors across 4 years was associated with risk of ovarian cancer similar to that found in the main analysis (fully adjusted model: high number of distress‐related factors, RR = 1.72, 95% CI = 1.06, 2.80; mixed number of factors, RR = 1.01, 95% CI = 0.83, 1.24).

**TABLE 2:** Risk of ovarian cancer by number of distress‐related psychosocial factors, Nurses' Health Study, Nurses' Health Study II, 1994–2015, *N* = 115,694.

Distress‐related psychosocial factors	Case/years	Model 1	Model 2	Model 3
		Hazard ratio (95% confidence interval)		
0	215/508,586	1.00 (Ref.)	1.00 (Ref.)	1.00 (Ref.)
1	200/458,189	1.01 (0.83, 1.23)	0.99 (0.82, 1.2)	0.99 (0.82, 1.2)
2	81/187,383	1.05 (0.81, 1.36)	1.03 (0.79, 1.34)	1.02 (0.79, 1.33)
3–4	30/39,770	1.70 (1.15, 2.50)**	1.65 (1.12, 2.44)*	1.62 (1.09, 2.42)*
Trend	526/1,193,927	1.09 (0.98, 1.22)	1.08 (0.97, 1.20)	1.08 (0.96, 1.20)

*Note*: Model 1 adjusted for age. Model 2 adjusted for oral contraceptive use, history of tubal ligation and hysterectomy, family history of ovarian cancer, duration and use of hormone replacement therapy, menopausal status, and parity. Model 3 further adjusted for physical activity, smoking status, and body mass index.

* *p* < 0.05

** *p* < 0.01.

Results were consistent in analyses stratified by menopausal status (Table S1). In sensitivity analyses examining high‐grade serous/poorly differentiated ovarian cancer as the outcome (*N* = 346 incident cases), women with ≥3 versus no indicators of distress were at greater risk for developing ovarian cancer (HR_age‐adjusted_ = 1.85; 95% CI = 1.17, 2.91; Table S2), further adjusted for ovarian cancer risk factors (HR = 1.82; 95% CI = 1.15, 2.88) and health behaviors (HR = 1.77; 95% CI = 1.10, 2.83).

### 3.1 PTSD subsample

Over follow‐up, 70 ovarian cancer cases occurred in the PTSD subsample. Replicating the main analysis in this subsample, women with ≥3 distress‐related factors were at 84% higher risk of ovarian cancer (RR = 1.84, 95% CI = 0.68–4.98) in the fully adjusted model, although this did not reach statistical significance with the limited cases (Table 3, Model A). With PTSD included as a distress‐related factor, having ≥3 distress‐related psychosocial factors versus none was associated with twofold greater ovarian cancer risk (HR = 2.08; 95% CI = 1.01, 4.29; Table 3, Model B). In further analyses with distress and PTSD considered separately, women with PTSD and any other distress‐related factor had the highest risk of ovarian cancer compared to the reference group of women with no PTSD and no other distress‐related factors (HR = 2.19, 95% CI = 1.20–4.01, Table 3, Model C). Results were similar in models including trauma exposure as a covariate and in analyses restricted to trauma‐exposed participants (Table 4, Models 3–4).

**TABLE 3:** Risk of ovarian cancer by number of distress‐related psychosocial factors, with and without posttraumatic stress disorder (PTSD) included as a measure of distress, restricted to participants with PTSD data, Nurses' Health Study II, 2003–2015, *N* = 38,530.

	Cases/person‐years	Hazard ratio (95% CI)
Model A: Depression, phobic anxiety, social isolation, and widowhood		
Distress‐related psychosocial factors		
0	24/137,458	1.00 (Ref)
1	22/141,514	0.85 (0.48, 1.52)
2	19/70,591	1.46 (0.80, 2.65)
3–4 factors	5/12,584	2.14 (0.81, 5.62)
Trend	70/362,147	1.26 (0.94, 1.68)
Model B: Depression, anxiety, social isolation, widowhood, and PTSD		
Distress‐related psychosocial factors		
0	21/122,854	1.00 (Ref)
1	14/125,915	0.63 (0.32, 1.25)
2	20/79,346	1.48 (0.78, 2.81)
3–5 factors	15/34,032	2.54 (1.28, 5.06)**
Trend	70/362,147	1.42 (1.09, 1.85)*
Model C: Depression, anxiety, social isolation, and widowhood, by PTSD status		
Distress‐related psychosocial factors		
No PTSD and no other factors	21/122,854	1.00 (Ref)
No PTSD and 1–4 other factors	22/167,878	0.73 (0.4, 1.33)
PTSD and no other factors	3/14,604	1.29 (0.38, 4.34)
PTSD and 1–4 other factors	24/56,810	2.41 (1.34, 4.34)**

*Note*: All models adjusted for age.

* *p* < 0.05

** *p* < 0.01.

**TABLE 4:** Risk of ovarian cancer by lifetime trauma exposure and number of distress‐related psychosocial factors, Nurses' Health Study II, *N* = 38,530.

	Case/years	Model 1: Trauma exposure	Model 2: Distress‐related factors	Model 3: Trauma and distress‐related factors	Model 4: Restricted to trauma‐exposed participants
	*N*	Hazard ratio (95% confidence interval)			
Lifetime trauma exposure					
No	15/79,820	1.00 (Ref)	–	1.00 (Ref)	–
Yes	55/282,327	0.99 (0.56, 1.74)		0.86 (0.48, 1.54)	
Distress‐related psychosocial factors					
0	21/123,276	–	1.00 (Ref)	1.00 (Ref)	1.00 (Ref)
1	14/126,139	–	0.60 (0.30, 1.20)	0.59 (0.30, 1.18)	0.42 (0.18, 0.99)*
2	21/79,165	–	1.38 (0.74, 2.60)	1.36 (0.73, 2.54)	1.21 (0.60, 2.41)
3–4	14/33,566	–	2.14 (1.06, 4.35)*	2.08 (1.04, 4.13)*	2.05 (0.98, 4.27)
Trend	70/328,581	–	1.35 (1.03, 1.76)*	1.33 (1.03, 1.73)*	1.36 (1.01, 1.83)*

*Note*: All models adjusted for oral contraceptive use, history of tubal ligation and hysterectomy, family history of ovarian cancer, duration and use of hormone replacement therapy, menopausal status, parity, physical activity, smoking status, and body mass index.

* *p* < 0.05.

## 4 DISCUSSION

Women experiencing three or more distress‐related psychosocial factors, including depression, social isolation, anxiety, and widowhood, were at more than 70% increased risk for ovarian cancer incidence than women experiencing none of these. This risk estimate is higher than previous reports of the association of any of these factors individually with ovarian cancer.^9^, ^11^, ^13^ Similar to prior findings, adjusting for health behaviors and ovarian cancer risk factors had a minimal impact on risk estimates, suggesting that this association is independent of these exposures. Results were consistent or slightly stronger when restricted to Type 2 ovarian cancer cases, which is important since many known ovarian cancer risk factors are only weakly associated with this histotype.^30^, ^31^, ^32^

In the subsample of women for whom PTSD was assessed, the association of number of distress‐related factors with ovarian cancer was substantially stronger when PTSD was included in the score. Women with PTSD and any additional distress‐related factors were at greater than twofold elevated risk; women with other forms of distress but without PTSD were not at elevated ovarian cancer risk. However, it is important to note the limited number of cases in this subsample, and the high level of co‐occurrence of PTSD with other forms of distress, which may reduce the ability to detect modest associations. We speculate that other forms of distress may confer risk, albeit with somewhat less magnitude relative to PTSD, perhaps because they have a broader range of severity and chronicity. Further, when PTSD occurs with other distress‐related factors, such as depression or history of childhood trauma, the resulting distress is likely to be more chronic and severe,^33^, ^34^ thus potentially having a greater impact on carcinogenic processes. It is possible that PTSD indicates higher or more consistent levels of vigilance, distress, and related biological dysregulation, compared with other distress‐related factors. Future work with appropriate data is needed to test these hypotheses.

Overall, our data support the hypothesis that having multiple distress‐related psychosocial factors is associated with increased risk of ovarian cancer incidence, consistent with findings that multiple stressful events^35^ or comorbid mental health conditions are associated with worse health outcomes.^36^, ^37^, ^38^, ^39^ Our findings further suggest that relative to other distress‐related factors, PTSD may be a particularly potent risk factor, which is consistent with our results when examining each distress factor individually.

Distress‐related factors may increase risk of ovarian cancer primarily via alterations in key stress‐related biological pathways; it is possible that individuals with multiple distress factors have more consistent or substantial dysregulation of such pathways. Specifically, social isolation, depression, anxiety, and PTSD have all been associated with higher concentrations of epinephrine and norepinephrine,^40^, ^41^, ^42^, ^43^ and some evidence suggests that these associations are stronger in persons with co‐occurring distress‐related factors.^44^ These hormones have been implicated in promoting tumor initiation, growth, metastasis, and invasiveness, and reduced survival.^2^, ^3^, ^5^, ^45^, ^46^, ^47^, ^48^, ^49^ Norepinephrine can induce DNA strand breaks and inhibit DNA repair, factors particularly critical in the development of high‐grade serous carcinoma.^5^, ^45^, ^46^ Norepinephrine has been associated with disruption of apoptosis and lower levels of anoikis (a process by which cells enter apoptosis after separation from neighboring cells and the extracellular matrix) in several cancers, including ovarian.^49^ Norepinephrine treatment of ovarian cancer cells increases expression of *DUSP1*, a phosphatase related to preventing apoptosis in cancer cell lines.^50^ Notably, a key early transcriptional response to norepinephrine is upregulation of *DUSP1* in normal immortalized cell lines from both ovarian and fallopian tube surface epithelium.^51^ Thus, both biologic and epidemiological studies support a role for distress‐related factors and chronic norepinephrine activation in the development and progression of ovarian cancer.

Our study has several limitations. Our sample included primarily White professional women; thus, generalizability may be limited, and levels of distress‐related psychosocial factors may be somewhat lower in this high functioning group compared to the general population. Indicators of distress were measured intermittently across follow‐up, and PTSD symptoms were queried only in a subset of women, retrospectively in 2008, potentially introducing some misclassification and reducing power in the analysis including PTSD. However, in this sample social isolation and anxiety were fairly stable across follow‐up (social isolation, intraclass correlation coefficient [ICC] = 0.68,^52^ anxiety, ICC = 0.66).^53^ Some common forms of distress linked to increased risk of disease outcomes in other work, such as loneliness or anger, were not queried. Our study also has important strengths. Multiple indicators of distress were queried prospectively and repeatedly across decades of follow‐up in a large and well‐characterized cohort. Presence and type of ovarian cancer were confirmed with medical record review, and many ovarian cancer risk factors and distress‐related health behaviors were queried throughout follow‐up, allowing for statistical adjustment.

## 5 CONCLUSIONS

We found that the presence of multiple indicators of distress was associated with greater risk of ovarian cancer than having a single indicator, and that PTSD may be the most strongly associated distress‐related ovarian cancer risk factor. This finding may help identify women at high risk for whom interventions to reduce distress might be beneficial beyond the mental health benefits and may also suggest that shared biological pathways across different types of distress are involved in ovarian cancer development. Our findings also support the need to simultaneously consider multiple distress‐related psychosocial factors. Overall, our findings add important evidence that the association of distress with ovarian cancer incidence and progression may be causal, due to the dose‐dependent relationship we found and careful accounting for known behavioral and ovarian cancer risk factors. Multiple forms of distress in women may not only indicate poor mental health but also indicate risk of cancer and other potentially fatal outcomes.^54^ Future research should determine whether preventing, mitigating, and treating distress could reduce subsequent poor health outcomes. Our findings may ultimately be important for clinical practice, to improve risk‐prediction models to identify high‐risk women who could benefit from preventive measures, such as opportunistic bilateral salpingectomy as an alternative to tubal ligation or during hysterectomy.

## AUTHOR CONTRIBUTIONS

**Andrea L. Roberts:** Data curation (lead); formal analysis (lead); methodology (equal); writing – original draft (lead). **Andrew Ratanatharathorn:** Formal analysis (equal); investigation (equal); methodology (equal); writing – review and editing (equal). **Lori Chibnik:** Formal analysis (equal); investigation (equal); methodology (equal); writing – review and editing (equal). **Laura Kubzansky:** Conceptualization (equal); data curation (equal); methodology (equal); supervision (equal); writing – review and editing (equal). **Shelley Tworoger:** Conceptualization (equal); funding acquisition (equal); investigation (equal); methodology (equal); supervision (equal); writing – review and editing (equal).

## FUNDING INFORMATION

This research was supported by National Institutes of Health grants UM1 CA186107, P01 CA87969, and U01 CA176726, and US Department of Defense grant OC120441. The funders had no role in the conceptualization, drafting, or approval of the manuscript or its contents.

## CONFLICT OF INTEREST STATEMENT

The authors declare no potential conflicts of interest.

## PARTICIPANT CONSENT

The study protocol was approved by the institutional review boards of the Brigham and Women's Hospital and Harvard T.H. Chan School of Public Health, and those of participating registries as required. Return of questionnaires by mail constituted implied consent. Signed releases were obtained to collect medical records.

## Supporting information


**Table S1.** **Table S2**.

## Data Availability

Data are available upon reasonable written request. According to standard controlled access procedures, applications to use NHS data will be reviewed for scientific aims, evaluation of the fit of the data for the proposed methodology, and verification that the proposed use meets the guidelines of the Ethics and Governance Framework and the consent that was provided by the participants.
