# Supplementary material for: Multiple types of distress are prospectively associated with increased risk of ovarian cancer
Source: Cancer Med. 2023 Jun 16;12(14):15404–13. doi: 10.1002/cam4.6125 (PMC10417295; doi:10.1002/cam4.6125)
Supplement: Supplementary file 1 — Table S1. Table S2. [file CAM4-12-15404-s001.docx]

**Supplemental Table 1.** Risk of ovarian cancer by number of distress-related psychosocial factors, stratified by menopausal status, Nurses’ Health Study and Nurses’ Health Study II

| **Premenopausal Women** | | | | |
| --- | --- | --- | --- | --- |
| **Distress-related psychosocial factors** | **Case/Years** | **Model 1** | **Model 2** | **Model 3** |
| 0 | 27 / 114,713 | 1.00 (Ref) | 1.00 (Ref) | 1.00 (Ref) |
| 1 | 21 / 106,911 | 0.84 (0.48, 1.50) | 0.81 (0.45, 1.45) | 0.82 (0.46, 1.48) |
| 2 | 12 / 49,436 | 0.96 (0.48, 1.93) | 0.90 (0.45, 1.82) | 0.88 (0.43, 1.78) |
| 3+ | 3 / 8,126 | 1.62 (0.49, 5.39) | 1.47 (0.43, 5.02) | 1.47 (0.42, 5.18) |
| Trend | 63 / 279,187 | 1.03 (0.75, 1.43) | 1.00 (0.72, 1.38) | 0.99 (0.71, 1.38) |
| **Postmenopausal Women** | | | | |
| 0 | 188 / 393,504 | 1.00 (Ref) | 1.00 (Ref) | 1.00 (Ref) |
| 1 | 179 / 350,724 | 1.04 (0.85, 1.28) | 1.02 (0.83, 1.25) | 1.02 (0.83, 1.25) |
| 2 | 69 / 137,676 | 1.06 (0.80, 1.41) | 1.05 (0.79, 1.39) | 1.04(0.78, 1.38) |
| 3+ | 27 / 31,606 | **1.73 (1.15, 2.60)** | **1.70 (1.12, 2.56)** | **1.67 (1.09, 2.54)** |
| Trend | 463 / 913,511 | 1.10 (0.98, 1.23) | 1.09 (0.98, 1.23) | 1.09 (0.97, 1.22) |

Model 1 adjusted for age. Model 2 adjusted for oral contraceptive use, history of tubal ligation and hysterectomy, family history of ovarian cancer, duration and use of hormone replacement therapy, menopausal status, and parity. Model 3 further adjusted for physical activity, smoking status, and BMI. Bolded estimates are statistically significant at p<0.05.

**Supplemental Table 2.** Risk of high-grade serous/poorly differentiated ovarian cancer by number of distress-related psychosocial factors, Nurses’ Health Study and Nurses’ Health Study II

| **Distress-related psychosocial factors** | **Case/Years** | **Model 1** | **Model 2** | **Model 3** |
| --- | --- | --- | --- | --- |
| 0 | 144 / 508528 | 1.00 (Reference) | 1.00 (Reference) | 1.00 (Reference) |
| 1 | 124 / 458073 | 0.95 (0.74, 1.21) | 0.93 (0.73, 1.18) | 0.93 (0.73, 1.18) |
| 2 | 56 / 187318 | 1.12 (0.81, 1.53) | 1.11 (0.81, 1.52) | 1.09 (0.80, 1.50) |
| 3+ | 22 / 39758 | **1.83 (1.16, 2.89)** | **1.79 (1.13, 2.83)** | **1.73 (1.08, 2.77)** |
| Trend | 346 / 1193677 | 1.12 (0.98, 1.28) | 1.111 (0.97, 1.27) | 1.10 (0.96, 1.26) |

Model 1 adjusted for age. Model 2 adjusted for oral contraceptive use, history of tubal ligation and hysterectomy, family history of ovarian cancer, duration and use of hormone replacement therapy, menopausal status, and parity. Model 3 further adjusted for physical activity, smoking status, and BMI. Bolded estimates are statistically significant at p<0.05.
